# Diarrhea-associated biofilm formed by enteroaggregative *Escherichia coli *and aggregative *Citrobacter freundii*: a consortium mediated by putative F pili

**DOI:** 10.1186/1471-2180-10-57

**Published:** 2010-02-23

**Authors:** Alex L Pereira, Thiago N Silva, Ana CMM Gomes, Ana CG Araújo, Loreny G Giugliano

**Affiliations:** 1 Laboratório de Microbiologia, Departamento de Biologia Celular, Universidade de Brasília, Brasília, DF, Brazil; 2 Embrapa Recursos Genéticos e Biotecnologia (Cenargen), Brasília, DF, Brazil

## Abstract

**Background:**

Enteroaggregative *Escherichia coli *(EAEC) are enteropathogenic strains identified by the aggregative adhesion (AA) pattern that share the capability to form biofilms. *Citrobacter freundii *is classically considered as an indigenous intestinal species that is sporadically associated with diarrhea.

**Results:**

During an epidemiologic study focusing on infantile diarrhea, aggregative *C. freundii *(EACF) and EAEC strains were concomitantly recovered from a severe case of mucous diarrhea. Thereby, the occurrence of synergic events involving these strains was investigated. Coinfection of HeLa cells with EACF and EAEC strains showed an 8-fold increase in the overall bacterial adhesion compared with single infections (P < 0.001). The synergic effect was mediated by physical interactions among the bacteria and primed in the absence of chemical signaling and without the participation of host cells. Thus, significant increases (2.7-fold on average) in bacterial adhesion were also observed during the formation of mixed biofilms on abiotic surfaces. Bacterial settling assays showed that EAEC strains harboring F-pili genes (*traA*) were capable of forming bacterial aggregates only in the presence of EACF. Scanning electronic microscopy analyses revealed that bacterial aggregates as well as enhanced biofilms formed by EACF and *traA*-positive EAEC were mediated by non-bundle forming, flexible pili. Moreover, mixed biofilms formed by EACF and *traA*-positive EAEC strains were significantly reduced using nonlethal concentration of zinc, a specific inhibitor of F pili. In addition, EAEC strains isolated from diarrheic children frequently produced single biofilms sensitive to zinc.

**Conclusions:**

Putative F pili expressed by EAEC strains boosted mixed biofilm formation when in the presence of aggregative *C. freundii*.

## Background

Enteroaggregative *E. coli *(EAEC) is defined by its adhesion to cultured cells in a stacked brick-like formation [1]. This phenotype, termed aggregative adherence (AA), is mediated by specific fimbriae (AAF) encoded by plasmids (pAA) [2,3]. However, other factors, including afimbrial adhesins, are also associated with this adherence pattern, indicating its complex nature [4,5].

The virulence of at least some prototype EAEC strains has been demonstrated [6], although it remains unclear whether virulent strains can be accurately screened in epidemiological studies. The gene sequence termed CVD432, found in pAA plasmids, has been employed as an EAEC molecular marker while the transcriptional activator termed AggR has been described as the major EAEC-virulence regulator [7]. Epidemiological studies suggest that CVD432-positive EAEC strains, which are predicted to carry the AggR regulon, form a subgroup of supposed pathogenic isolates named "typical EAEC" [8]. Despite the efforts to identify a genotype definitely associated with the EAEC virulence, controversial data gathered in different geographic areas has made the epidemiology of this pathotype difficult to understand. Nevertheless, EAEC has been recognized as an emerging pathogen mainly associated with persistent infantile diarrhea in middle-income countries [9,10].

Elucidation of the mechanisms involved in EAEC pathogenesis has been limited because of the heterogeneity displayed by wild-type strains [6,11]. Given this genetic heterogeneity, expression of biofilms has been considered a consensual virulence factor among EAEC isolates [1,12,13]. Biofilm formation is a complex event that may involve many species and several factors. Furthermore, the discovery that factors not devoted to adhesion are also important in biofilm formation has highlighted its multifactorial nature. An AAF-independent mechanism for biofilm formation, which is mediated by plasmid-encoded type IV pili, was described in the atypical EAEC strain C1096 [14].

Type IV pili are involved in numerous phenotypes in gram-negative pathogens including cell adhesion, twitching motility and conjugation [15,16]. In addition to type IV pili, *tra *gene-encoded pili are involved in bacterial conjugation mediated by F plasmids. These cellular appendages are non-bundle forming, flexible pili reaching 5 μm in length that are expressed during log phase [17-19]. Furthermore, F pili render planktonic bacteria capable of engaging in biofilm formation by allowing cell-to-cell contact and interactions with abiotic surfaces [20]. Thus, it has been shown that *E. coli *strains harboring natural F plasmids form complex mature biofilms by using F-pilus connections in initial stages of the biofilm formation, whereas plasmid-free strains form only patchy biofilms [21].

Bacteria that express conjugation systems frequently exhibited cell aggregation followed by flocculation in static liquid culture. In *E. coli *strains, bacterial autoaggregation is also mediated by the expression of the self-recongnizing adhesin named antigen 43 (Ag43). Ag43 is a autotransporter protein whose the mature form consists of two subunits, α and β [22]. The expression of Ag43 is phase variable and in the K12 strain is under the control of OxyR, the master activator of the oxidative stress response in *E. coli *strains [23]. In addition to Ag43, bacterial aggregation is also mediated by the expression of curli fibers. Curli is a proteinaceous component of the extracellular matrix produced by many *Enterobacteriaceae *species which is known as thin aggregative fimbriae [24]. Among *Enterobacteriacea *species, curli fibers are the major determinant of cell-cell interactions and adherence to abiotic surfaces and have been shown to sustain biofilm formation in *Enterobacter *sp., *Salmonella *Typhimurium, *E. coli *and *Citrobacter freundii *strains [25].

*Citrobacter freundii *is usually considered a commensal species of the human gut, although some isolates have acquired specific virulence traits that enable them to cause diarrhea. Therefore, virulence factors homologous, and some even identical, to those described in *E. coli *pathotypes were detected in *C. freundii *strains isolated from sporadic cases of infantile diarrhea [26-29]. Additionally, isolates of *C. freundii *have been identified as effective recipient strains even since the first articles concerning *E. coli *conjugation mediated by F pili were published [30]. Reports on the transfer of *E. coli *thermo-stable toxin genes between these species raised considerations about the virulence potential of the bacterial conjugation [29,31,32]. A highly conjugative plasmid (pCTX-M3), which is responsible for the extensive spread of extended-spectrum β-lactamase (ESBL) in *Enterobacteriaceae*, was described in clinical isolates of *C. freundii*. pCTX-M3 is a 89,468 bp-plasmid belonging to IncL/M group that probability evolved from environmental plasmids through stepwise integration of mobile genetic elements. Moreover, it has been shown that this plasmid is easily transferred to *E. coli*, *Klebsiella *sp., *Enterobacter cloacae*, *Serratia marcescens *and *Salmonella enterica *strains [33,34].

Nowadays, it is known that phenotypic features classically associated with pathogenic *E. coli *strains are not restricted exclusively to this species. In addition to EAEC, the AA pattern has been recognized in uropathogenic *Proteus mirabilis *strains [35] and in *Klebsiella pneumoniae *strains recovered from healthcare-associated infections [36]. In these isolates, the expression of AA pattern has been associated with the ability to form biofilms.

Bacterial biofilms found in natural and pathogenic ecosystems are formed in the presence of multiple species and genetically distinct strains. However, the current understanding of these microbial consortia is largely based on single-species models that frequently use laboratory strains. In this work, wild-type strains of typical EAEC and *C. freundii*, which were concomitantly recovered from diarrhea, were tested in mixed biofilm assays in order to evaluate the occurrence of synergistic effects on biofilm formation.

Firstly, it is shown that the diarrhea-isolated *C. freundii *strain shared the characteristic AA phenotype displayed by EAEC strains, and henceforth was named aggregative *C. freundii *(EACF). It follows that EACF strain 205 and diarrhea-isolated typical EAEC strains cooperate to increase bacterial adhesion to HeLa cells and biofilm formation. Moreover, the synergic effect was associated with putative F pili expressed by EAEC strains.

## Results

### Aggregative *C. freundii*

**Figure 1:**
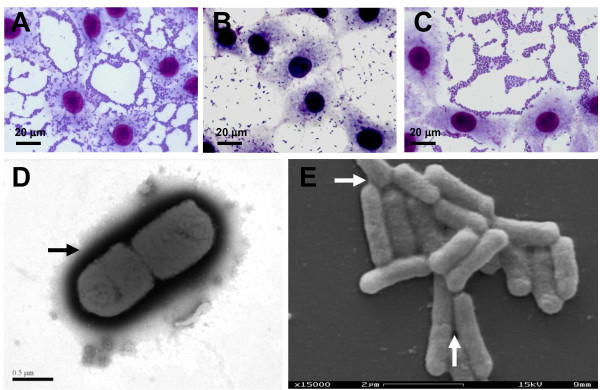
**Adhesion to HeLa cells and ultrastructural analyses of aggregative *C. freundii***. Micrographs A and B show the adherence pattern displayed by aggregative *C. freundii *205 (EACF 205) and diffusely adherent *C. freundii *047, respectively. For comparison, AA pattern displayed by prototype EAEC strain 042 is shown in the micrograph C. Electronic micrographs of EACF 205 are shown in the frames D and E. Both planktonic and surface-associated EACF cells did not displayed fimbrial structures; however, an extracellular matrix was detected surrounding the bacterial cells (arrows in frames D and E).

During a case-control study of infantile diarrhea, *C. freundii *strains were isolated from two subjects. The *C. freundii *strain 205 (Cf205) was isolated from a child suffering of a severe mucous diarrhea; while the strain 047 (Cf047) was isolated from a control child. Adherence assays showed that strain Cf205 displayed a mannose-resistant AA phenotype (Figure 1A) indistinguishable to that developed by EAEC prototype strain 042 (Figure 1C). As with the prototype EAEC strain, Cf205 strain displayed the characteristic stacked-brick pattern on the periphery of the cells and autoagglutination on the glass coverslip. Therefore, this strain was termed aggregative *C. freundii *(EACF). By contrast, control strain Cf047 developed diffuse adherence (Figure 1B).

Given the occurrence of aggregative adherence in *C. freundii*, the presence of EAEC adhesion related fimbrial genes together with 7 additional EAEC molecular markers were tested (Table 1). None of the EAEC-specific genetic markers were detected in the EACF strain and in the diffusely adherent strain as well. Additionally, eleven virulence markers associated with four other *E. coli *pathogenic categories were also tested and included markers for toxins and adhesins (Table 1). None of these tested markers were detected in the examined *C. freundii *strains. *C. freundii *strains were also tested negative for gene sequences of the self-recognizing adhesin Ag43.

**Table 1:** Primers used for detection of *E. coli *molecular markers

Gene	Locus description	Primer sequence (5'-3')	Amplicon length (bp)	Annealing temperature (°C)	Reference
**Enteroaggregative *E. coli *markers**					
*aat*	AA probe (CVD432)	CTGGCGAAAGACTGTATCAT	630	55-60	[9]
		CCATGTATAGAAATCCGCTGTT			
					
*aggR*	Transcriptional activator	CTAATTGTACAATCGATGTA	324	50	This study
		CTGAAGTAATTCTTGAAT			
					
*aggA*	Aggregative fimbria I (AAF I)	GCTAACGCTGCGTTAGAAAGACC	421	55-60	[9]
		GGAGTATCATTCTATATTCGCC			
					
*aafA*	AAF/II	GACAACCGCAACGCTGCGCTG	233	50	[9]
		GATAGCCGGTGTAATTGAGCC			
					
*agg3A*	AAF/III	GTATCATTGCGAGTCTGGTATTCAG	462	60	[5]
		GGGCTGTTATAGAGTAACTTCCAG			
					
*pilS*	Type IV pilus	ATGAGCGTCATAACCTGTTC	532	58	[14]
		CTGTTGGTTTCCAGTTTGAT			
					
*pic*	Mucinase	TTCAGCGGAAAGACGAA	500	55-60	[9]
		TCTGCGCATTCATACCA			
					
*pet*	Plasmid-encoded toxin	CCGCAAATGGAGCTGCAAC	1,133	55-60	[9]
		CGAGTTTTCCGCCGTTTTC			
					
*astA*	EAEC heat-stable toxin	CCATCAACACAGTATATCCGA	111	55-60	[9]
		GGTCGCGAGTGACGGCTTTGT			

**Enteropathogenic *E. coli *markers**					
*EAF *probe	EPEC adhesion factor	CAGGGTAAAAGAAAGATGATAA	396	52	[9]
		TATGGGGACCATGTATTATCA			
					
*eae*	Intimin (adhesin)	CCCGAATTCGGCACAAGCATAAGC	877	52	[9]
		CCCGGATCCGTCTCGCCAGTATTCG			
					
*escC*	Locus of enterocyte effacement (LEE) 2	GTCAGCGACAGATATAACATAC	450	54	[44]
		AACGCATTCACCCTAATC			
					
*escV*	LEE 3	CTAACTTCTTTCCCCACAATC	760	54	[44]
		TATCCCCAACAGGCAAAC			

**Enterohaemorrhagic *E.coli *markers**					
*stx*	Shiga toxin I and II	TTTACGATAGACTTCTCGAC	227	48	[45]
		CACATATAAATTATTTCGCTC			
					
*hlyA*	hemolysin	GGTGCAGCAGAAAAAGTTGTAG	1,551	57	[46]
		TCTCGCCTGATAGTGTTTGGTA			

**Enterotoxigenic *E. coli *markers**					
*cfaA-B*	Colonization factor antigen 1	CTATTGGTGCAATGGCTCTGACC	352	55-60	[47]
		GCAGCAGCTTCAAATTCTTTGGC			
					
*cs3*	Colonization factor CS3	CCACTCTAACCAAAGAACTGGC	250	60	This study
		GGTGGTGGCAAAGCTAGCAGAG			
					
*ltA*	Heat-labile enterotoxin	GGCGACAGATTATACCGTGC	696	50	This study
		CCGAATTCTGTTATATATGTC			
					
*estA*	Heat-stable enterotoxin	CAGGATGCTAAACCAGTAGAGT	174	60	This study
		TCCCTTTATATTATTAATAGCACCC			

**Uropathogenic *E. coli *markers**					
*papC*	P pili usher	GACGGCTGTACTGCAGGGTGTGGCG	328	60	[48]
		ATATCCTTTCTGCAGGGATGCAATA			
					
*sfaD-E*	S fimbria	CTCCGGAGAACTGGGTGCATCTTAC	407	60	[48]
		CGGAGGAGTAATTACAAACCTGGCA			

As conjugation may lead to bacterial aggregation, the presence of conjugative plasmids was also tested employing primers designed to target pCTX-like plasmids (*traJ *primers) and F plasmids (*traA *primers). *C. freundii *strains were negative for the tested conjugative sequences. Moreover, plasmid profile revealed that EACF and diffusely *C. freundii *were plasmid-free strains (data not shown).

In an attempt to reveal some aspect on the adhesion factor used by the EACF strain, ultrastructural analyses were carried out. TEM micrographs showed that planktonic cells of EACF did not display fimbrial structures (Figure 1D). EACF biofilms were also analyzed using scanning electron microscopy. Surface-associated EACF cells formed tight aggregates which were devoid of extracellular appendages (Figure 1E). Although extracellular appendages can not be detected in the EACF strain, the presence of an extracellular matrix involving both planktonic (Figure 1D) and surface-associated (Figure 1E) EACF cells was easily noted. Together these results indicated the occurrence of putative non-fimbrial adhesins mediating the adhesion of the EACF strain.

### EACF 205 and EAEC strains cooperate to increase adhesion to HeLa cells

**Figure 2:**
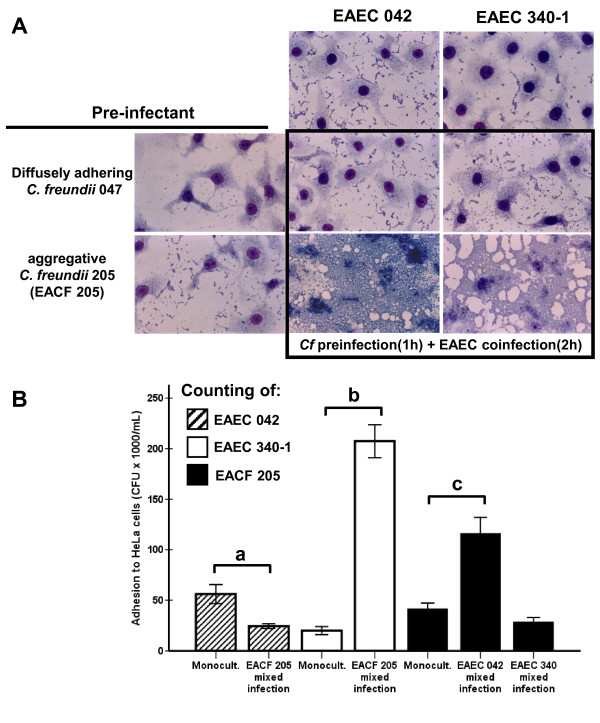
**Mixed infection assays**. A- Qualitative assay. Aggregative *C. freundii *(EACF) strain 205 improves bacterial adhesion when in combination with typical EAEC strains. B- Quantitative mixed infection assay. Adherence to HeLa cells displayed by EACF 205 and EAEC strains in mixed infections assays was quantified using the counting of colony-forming units (CFU), and was compared with adhesion displayed by the monocultures. EAEC strains showed antagonistic behaviors when in presence of EACF 205. ^a ^denotes *P *< 0.05 for comparison of 2 groups; ^b and c ^*P *< 0.001. Statistical analyses: independent-sample T test.

Aware that EACF strain 205 was isolated from a severe diarrhea case together with EAEC strains, mixed infection assays were conducted in order to evaluate the adherence developed by bacterial combinations (*C. freundii *and EAEC) recovered from the diarrheic child 205 and from the healthy child 047. Light microscopy showed that the adhesion to HeLa cells developed by the pair of strains isolated from diarrheic child (EACF 205 plus EAEC 205-1) was greater than that supported by each of the strains separately as well as by the bacterial pair recovered from control child (*C. freundii *047 plus EAEC 047-1). As both EAEC strains 205-1 and 047-1 showed cell-detaching activity, they are considered unsuitable for quantitative analyses. Therefore, the diarrhea-isolated EAEC strain 340-1 and the prototype EAEC strain 042 were chosen in order to continue the mixed infection assays employing quantitative analyses. As verified in the preliminary tests, the preinfection of HeLa cells with EACF strain 205 increased the bacterial adherence when followed by coinfection with EAEC strains 340-1 or 042 (Figure 2A). In contrast, preinfection with control-isolated *C. freundii *strain 047 did not cause any increment of bacterial adhesion.

To exclude the possibility that the increased adhesion was an unspecific synergic effect triggered by any pair of aggregative strains, coinfection assays were performed with several pairs of EAEC strains (EAEC 340-1 and EAEC 042; EAEC 205-1 and EAEC 042; EAEC 340-1 and EAEC 205-1). No increment in bacterial adhesion was observed using any strain combination.

In order to determine what species accounted for the increased adhesion, quantitative mixed infection assays were conducted and the colony forming units (CFU) were counted (Figure 2B). Assays showed that EAEC strains 340-1 and 042 displayed antagonistic behaviors when HeLa cells were preinfected with EACF strain 205. Regarding EAEC 340-1, preinfection with EACF 205 induced a 10-fold increase in the adherence of strain 340-1 when compared with the single infection (*P *< 0.001). By contrast, preinfection with EACF 205 decreased adhesion of the EAEC strain 042 at 43.5% (*P *< 0.05). The overall increased adhesion displayed by coinfection of EACF 205 plus EAEC 042 was supported by the 2.8-fold increased adherence of the EACF 205 (*P *< 0.001).

### Search for biochemical signaling

The role of inter-specific chemical signals in the increase of bacterial adherence was evaluated using permeable inserts that allow the division of culture-plate wells into two diffusion chambers. Thus, DMEM media were pre-conditioned inoculating the upper chamber with bacterial cultures, and then HeLa cells, in the lower chamber, were infected in order to test the bacterial adherence. Media pre-conditioned by EACF 205 or by EAEC strains did not induce changes in the adhesion developed by EAEC 340-1, EAEC 042 or EACF 205. Such results indicated that the increase in adherence was not triggered by chemical signaling.

**Figure 3:**
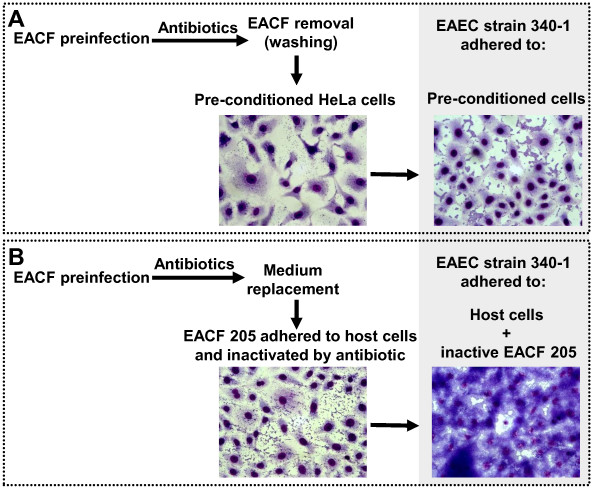
**Adhesion of EAEC strain 340-1 to pre-conditioned HeLa cells**. Frame A describes the adhesion assay employing host cells pre-conditioned by the adherence of EACF strain 205. Frame B shows the parallel assay that was carried out in the absence of washing step. Bacterial cells of EACF 205 adhered to HeLa cells and inactivated by antibiotics still held the capability to boost EAEC adherence.

The possibility that a previous intimate bacterial contact with the host cells might be required for the increment in adherence was also tested. HeLa cells pre-conditioned by the adhesion of EACF 205 were treated with antibiotics and washed in order to remove the adherent bacteria. Afterwards, pre-conditioned HeLa cells were used to test the adhesion of the EAEC strains (Figure 3, frame A). No increment in bacterial adherence was observed showing that the enhanced adhesion was not primed by host cells. However, the same assay carried out in the absence of washing step showed an increased adherence similar to that observed with live bacteria. Thus, the EACF 205 population adhered to HeLa cells and inactivated by antibiotics still held the capability to boost the adhesion of the EAEC strain 340-1 (Figure 3, frame B). These results showed that the increase in the bacterial adherence developed by EACF 205-EAEC combinations were supported by physical interactions, which were triggered by EAEC strains, independently of chemical signals or the influence of host cells.

### EACF 205 and *traA*-positive EAEC strains form bacterial aggregates

Aggregation assays showed that the EAEC strain 042 was capable of intense autoaggregation (aggregation rate of 0.999 ± 0.007). As a consequence, this strain was not used in the aggregation assays which intended to address inter-specific interactions. Standing overnight cocultures of EACF 205 and EAEC 340-1 aggregated at levels (0.70 ± 0.04) higher than *C. freundii *047-EAEC 340-1 cocultures (0.52 ± 0.05) and monocultures of EACF 205 (0.34 ± 0.11), *C. freundii *047 (0.12 ± 0.02) or EAEC 340-1 (0.53 ± 0.05). These assays indicated the occurrence of inter-specific interactions between EACF 205 and EAEC 340-1. Settling profile assays showed that the bacterial aggregates formed by EACF 205 and EAEC 340-1 were not restored if the overnight coculture was homogenized. Moreover, the assays showed that bacterial aggregates were not formed when overnight monocultures of EACF 205 and EAEC 340-1 were mixed (data not shown). These results indicated that the aggregation involving EACF 205 and EAEC 340-1 strains occurred at a specific time during the bacterial growth and involved inter-specific recognition.

**Figure 4:**
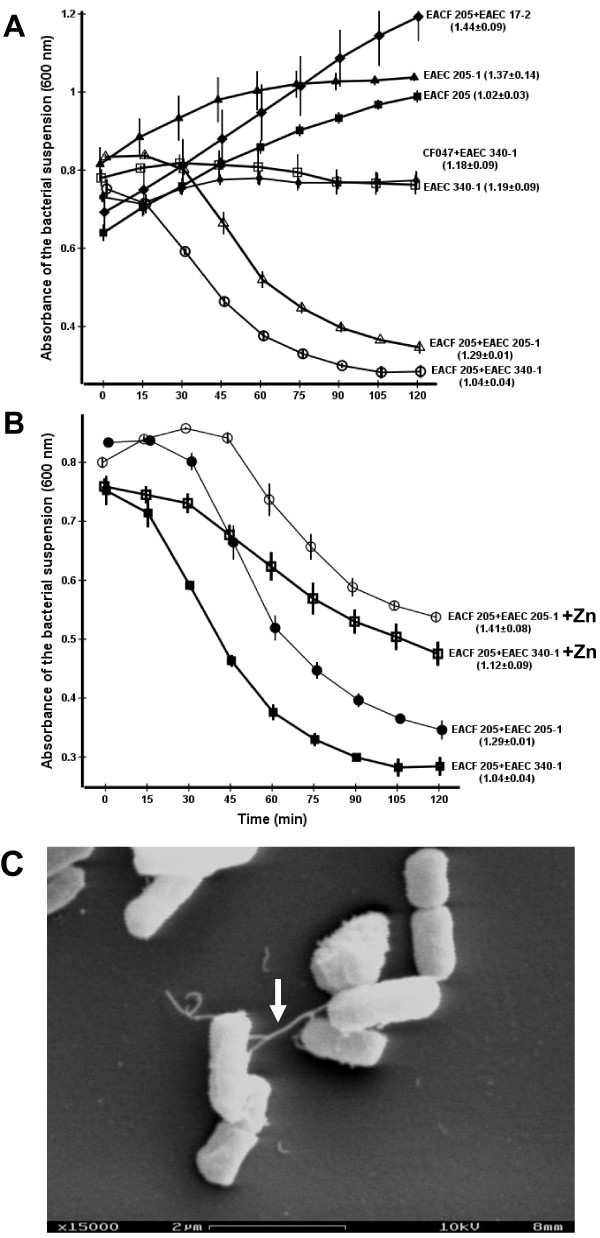
**Settling profile assays**. The numbers in parentheses indicate the final optical density of the bacterial suspension after homogenization. A- Settling profile displayed by EACF 205 and EAEC strains. Bacterial aggregates were formed only when EACF 205 was mixed with *traA*-positive EAEC strain 340-1 or 205-1. B- Effect of zinc on the settling kinetic developed by EAEC strain 340-1 or 205-1 in the presence of EACF 205. C- SEM micrograph showing non-bundle forming, flexible pili (white arrow) mediating the formation of EACF-EAEC aggregates. Pili extend away from bacteria up to 2 μm, connecting other bacteria.

In order to verify these events, settling profile assays were performed employing bacterial cultures in mid-log phase. The assays showed that EAEC strains 340-1 and 205-1 aggregated, and consequently settled, only in the presence of EACF 205 (Figure 4A). When mixed with EACF 205, the EAEC strains 340-1 or 205-1 induced a steady drop in the settling curve at the 15-min time point. Aggregation occurred in the absence of self-recognition, given that monocultures of tested EAEC strains and EACF strain 205 did not settle. SEM analyses showed that bacterial aggregates were mediated by non-bundle forming, flexible pili that extended up to 2 μm and promoted cell-to-cell contact (Figure 4C). By contrast, EACF 205 was unable to aggregate when combined with EAEC strain 17-2, demonstrating the absence of inter-specific interactions between these strains (Figure 4A). Confirming this fact, SEM analyses did not detect any bacterial appendages in the mixed suspensions of EACF 205 and EAEC 17-2.

The inter-specific recognition mediated by flexible pili during the mid-log phase indicated the involvement of conjugative pili in the formation of the bacterial aggregates [17,18]. Endorsing this assumption, EAEC strains 340-1 and 205-1 were shown to harbor *traA *family genes. In contrast, the EAEC 17-2, which was unable to display inter-specific aggregation with EACF 205, was negative for *traA *genes. Further evidence was obtained employing zinc, a F-pili specific inhibitor. The zinc treatment of the EAEC strain 340-1 or 205-1 impacted negatively the respective settling curves when performed in the presence of EACF 205 (Figure 4B). Magnesium, another divalent ion which was used in control assays, did not inhibit the bacterial aggregation (data not shown).

### AAF-positive EAEC strains harboring the *traA *gene boosted mixed biofilm formation

In the search for the presence of potential adherence factors listed in table 1, with the exception of the locus *tra*, the EAEC strains 17-2 (*traA*-), 340-1 (*traA*+) and 205-1 (*traA*+) shared the same genotype: pCVD432^+^AggR^+^AAF-I^+^PilS^+^Pap^+^. These strains were therefore employed to verify the association of the *traA *gene with the increase in biofilm formation in EACF-EAEC cocultures.

**Figure 5:**
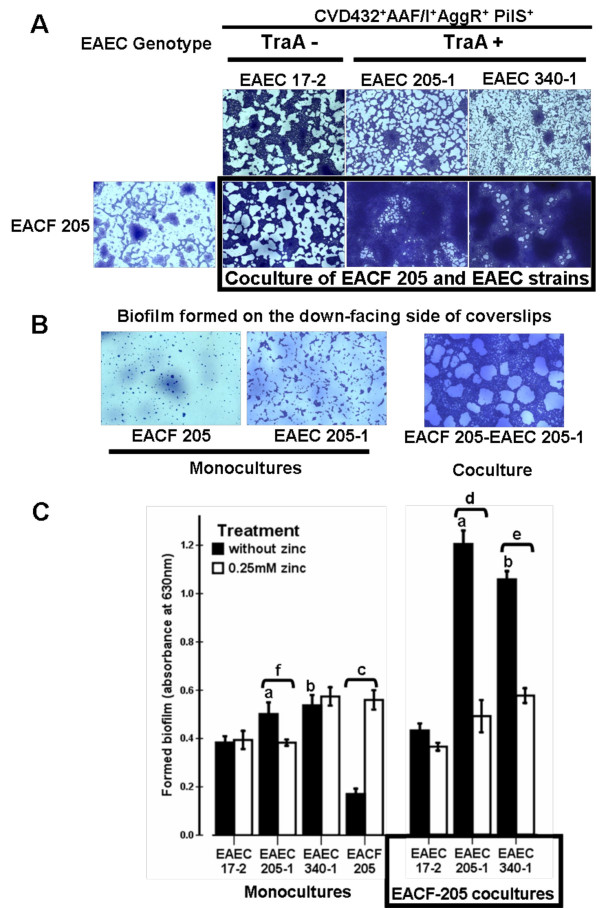
**Biofilm formation on glass coverslips**. A- Micrographs showing the upper-facing side of the glass coverslips. Biofilms formed by EACF 205 or by EAEC strains were compared with mixed biofilms produced by cocultures of EACF 205 and EAEC strains. EAEC genotype denotes the specific combination of EAEC markers hosted by *E. coli *strains. Enhanced biofilms were formed by the coculture of EACF 205 and *traA*-positive EAEC strains. B- Micrographs showing the down-facing side of the glass coverslips. Enhanced biofilms formed by the coculture of EACF 205 and *traA*-positive EAEC strains indicating an active processes rather than a mere fate following the bacterial settling. C- Quantitative assays. ^a, b, c, d and e ^denote *P *< 0.001 for comparison of 2 groups; ^f ^*P *< 0.05. Statistical analyses: independent-sample T test.

Preliminary assays showed that the synergic effect, previously detected using HeLa cells, was reproducible when glass coverslips were used as adhesion substratum (Figure 5A). The increased adhesion occurred in both faces of the coverslips indicating that enhanced biofilms were caused by active processes developed by combination of EACF 205 and *traA*-positive EAEC strains rather than a mere consequence of bacterial settling (Figure 5B). Mixed biofilms formed by cocultures of EACF 205 and *traA*-positive EAEC strains (340-1 or 205-1) were 2.7-fold increased when compared with single biofilms supported by each respective EAEC strain (*P *< 0.001) (Figures 5A and 5C). In contrast, the mixed biofilm developed by EACF 205 and EAEC 17-2 (*traA*-negative strain) (OD 0.431 ± 0.084) did not display a statistically significant increase when compared with the EAEC 17-2 single biofilm (OD 0.383 ± 0.079) (*P *= 0.237) (Figures 5A and 5C).

### Zinc effect on single and mixed biofilms

Single and mixed biofilm assays were performed in order to evaluate the impact of zinc, and consequently the role of putative F pili, on biofilm formation (Figure 5C). Zinc at a concentration of 0.25 mM (12-fold lower than zinc MIC - minimum inhibitory concentration) reduced the single biofilm formation by EAEC strain 205-1 by 23% (*P *= 0.038) (Figure 5C). In the case of EAEC strains 340-1 and 17-2 no reduction in single biofilms was noted. In contrast, the single biofilm formed by EACF 205 displayed a 3-fold increase when zinc was present (*P *< 0.001) (Figure 5C). Focusing on the *traA*-positive EAEC strains, these results indicate that putative F pili assume variable relevance in the formation of single biofilms.

The impact of zinc on mixed biofilm developed by cocultures of EACF 205 and EAEC strains was also evaluated. Zinc significantly reduced (*P *< 0.001) EACF-205 mixed biofilms formed by EAEC 205-1 (59%) or by EAEC 340-1 (45%) which displayed in these conditions similar levels to those reached by EACF 205 single biofilms (Figure 5C). As expected, zinc treatment did not impact the mixed biofilm produced by EACF 205 and EAEC 17-2 (*traA*-negative strain) endorsing the conclusion that this biofilm was formed in the absence of putative F pili.

Taken together, these results indicated that putative F pili engaged EAEC strains in mixed biofilm formation when EACF was present.

### SEM analyses of biofilms

**Figure 6:**
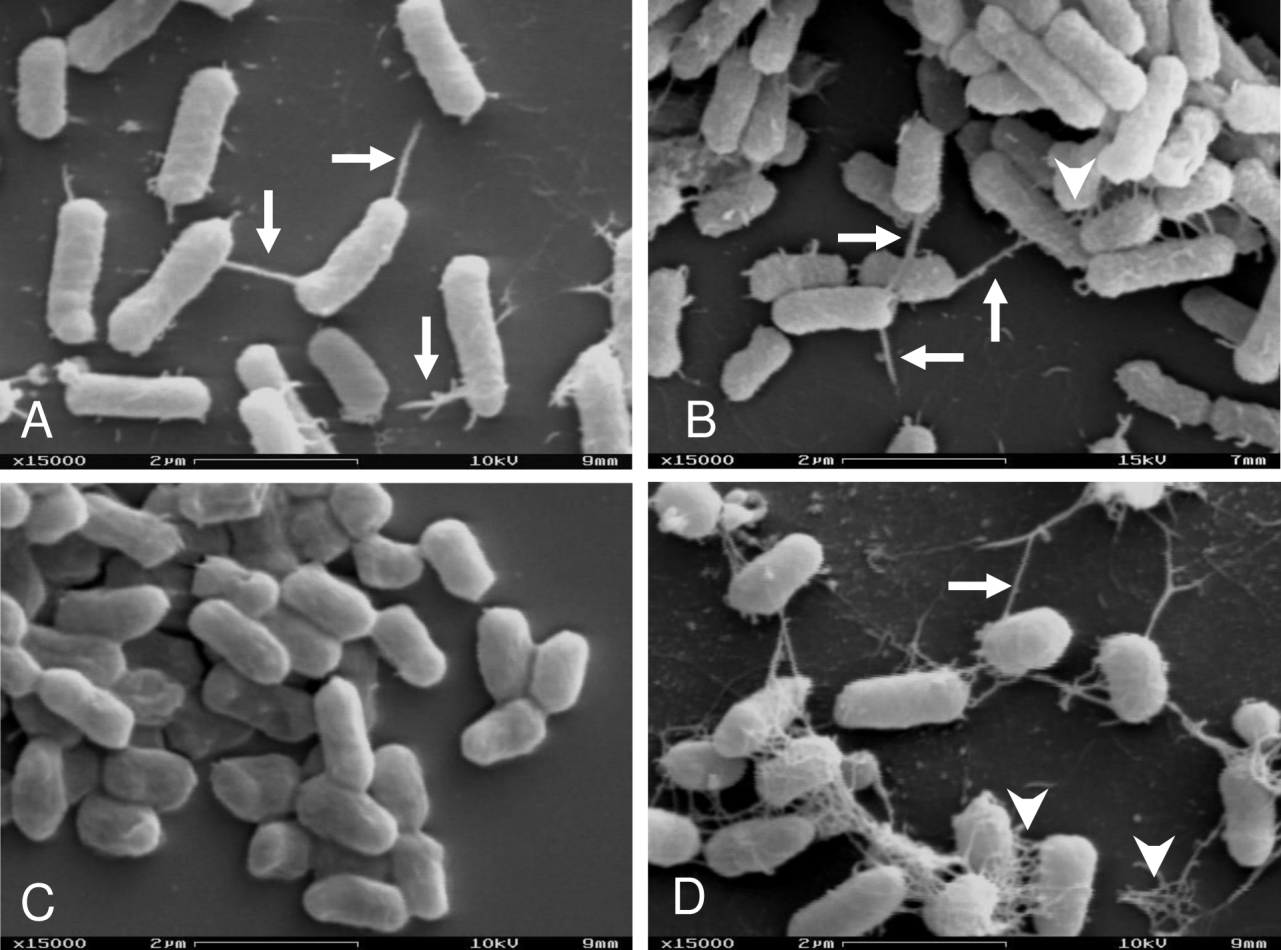
**SEM micrographs showing the biofilms developed by EACF 205 and EAEC strains**. A- Single biofilm formed by *traA*-positive EAEC strain 340-1. Arrows indicate the putative F pili. Note that pili were not limited to the polar region of the bacteria and, at times, were viewed to intertwine forming thicker structures. B- Enhanced biofilm developed by coculture of EACF 205 and *traA*-positive EAEC strain 340-1. White arrowhead indicates the incipient formation of curli fibers and arrows indicate the putative F pili. C- Single biofilm developed by *traA*-negative prototype strain 17-2. D- Single biofilm formed by EAEC 340-1 displaying curli fibers (white arrowheads). Curli fibers were shown to mediate cell-cell adherence and interaction to abiotic surface. Arrow indicates a putative F pilus.

SEM micrographs showed that EACF-205 biofilms occurred in the absence of any extracellular appendage (Figure 1E). By contrast, biofilms formed by EAEC strains 340-1 or 205-1 were mediated by thick pili that emanated from bacteria and regularly attached to the abiotic surface (Figure 6A). Enhanced biofilms developed by cocultures of EACF 205 with EAEC strains 340-1 or 205-1 were also mediated by pili that, in addition to adhesion to inert surfaces, frequently promoted cell-to-cell interactions (Figure 6B). As expected, putative F pili were not detected in the single biofilms formed by *traA*-negative EAEC strain 17-2 (Figure 6C). Curli fibers were occasionally detected in biofilms formed by EAEC strain 340-1 mainly during single biofilm formation (Figure 6D).

### Zinc effect on single biofilms produced by typical EAEC strains isolated from asymptomatic and diarrheic children

**Figure 7:**
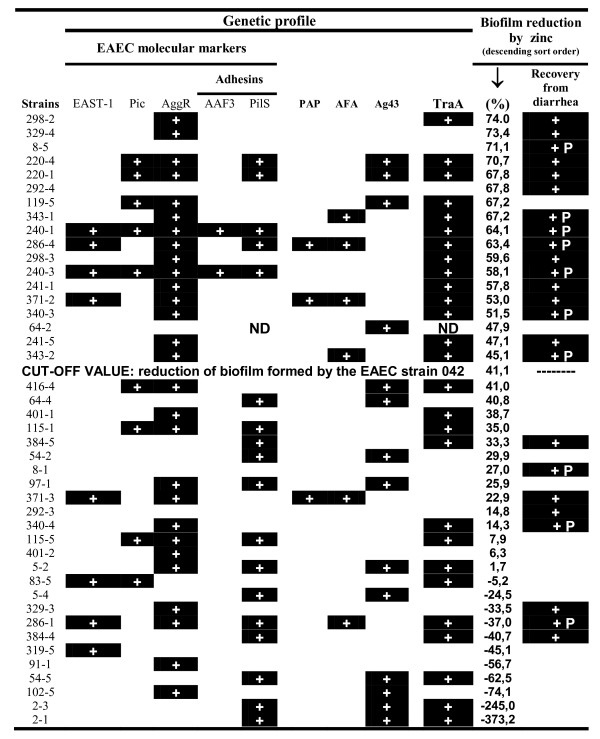
**Characterization of the typical EAEC strains which were tested for biofilm sensitivity to zinc**. Most of the strains isolated from diarrhea positioned above the cut-off value and thus were considered to form biofilms sensitive to zinc. **Abbreviations**: **EAST-1 **(thermo-stable toxin of EAEC), **Pic **(protein involved in colonization), **AggR **(transcriptional activator of EAEC), **AAF3 **(aggregative adherence fimbria III), **PilS **(type IV pilus of EAEC), **PAP **(pili P), **AFA **(Afa-Dr operon), **Agn43 **(antigen 43), **TraA **(pilin F), **+ **(positive result) **P **(persistent diarrhea - lasting more than 14 days), **ND **(not determined).

**Figure 8:**
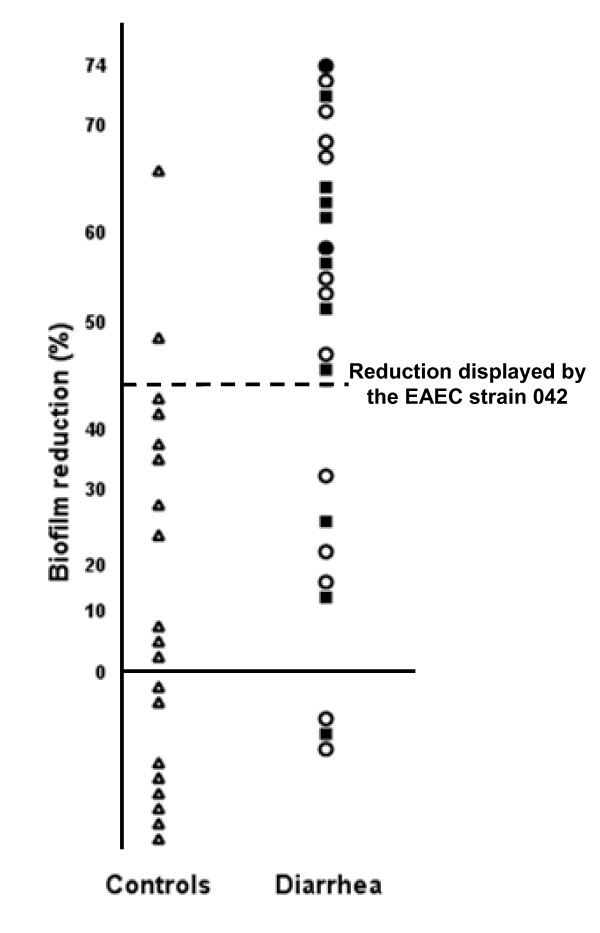
**Effect of the zinc on the biofilms formed by typical EAEC strains isolated from children with diarrhea and controls**. The data represent the average percent reduction in biofilm formation obtained after three independent assays at least. Solid squares represent the reduction displayed by strains recovered from persistent diarrhea (> 14 days); solid circles represent the reduction displayed by strains recovered from diarrhea lasting 12 days; and open circles represent the reduction displayed by strains isolated from diarrhea lasting 10 days or less. Reductions in biofilm formation displayed by strains isolated from healthy children are represented by triangles. Dotted line indicates the average reduction displayed by the prototype EAEC strain 042.

In order to evaluate the role of putative F pili on biofilm formation, 43 AAF (I and II)-negative EAEC strains, including 24 strains recovered from diarrhea and 19 recovered from healthy children (control group), had their ability to form biofilms challenged by zinc. Additional genetic characterization (Table 1) showed that two of these strains were positive for AAF/III and that six strains harbored adhesion factors associated with other *E. coli *pathotypes (Figure 7). Employing the average reduction presented by *traA*-positive EAEC prototype strain 042 (41.1%) as a cut-off line, the assays showed that the EAEC strains were sorted into two groups plotted in opposite positions (Figure 8). Most of the strains isolated from diarrhea positioned above the cut-off line and thus were considered to form biofilms sensitive to zinc. Specifically, sixteen of 24 (66%) diarrhea-isolated strains were ranked above the cut-off line. In addition, seven of 10 strains recovered from persistent diarrhea formed biofilms sensitive to zinc (*P *< 0.01 comparing with control group). In contrast, 17 of 19 (89%) strains isolated from healthy children formed biofilms resistant to zinc (*P *< 0.001 when compared with diarrheic group).

This approach suggested that typical EAEC strains use distinct adherence factors to form biofilms. Moreover, the assays showed that most of EAEC recovered from diarrhea employ putative F pili as central factors during biofilm formation. On the other hand, EAEC strains isolated from controls commonly use zinc-resistant adhesins to form biofilms.

Despite of the genetic heterogeneity presented by the tested collection of typical EAEC strains (Figure 7), the zinc cut-off line showed a specificity of 89.4% and predictive positive value of 88.9% when employed as a sorting criterion for diarrhea-associated typical EAEC strains.

## Discussion

Despite controversial data gathered from different geographic areas, epidemiological studies conducted in economically underprivileged communities showed that EAEC strains are strongly associated with persistent diarrhea in children [9]. EAEC is also associated with growth impairment caused by malabsorption that, theoretically, would occur in consequence of thick biofilm formation [37]. Moreover, it has been suggested that unrecognized enteropathogens might be involved in similar pathologic processes [10,37]. This work showed that EACF 205 boosted the bacterial adhesion to HeLa cells as well as the biofilm formation when in the presence of typical EAEC strains. Despite the antagonistic behavior displayed by EAEC strains 340-1 (increased adhesion) and 042 (decreased adhesion) when in the presence of EACF 205 (Figure 2B), the overall bacterial adhesion was always increased in the mixed infection assays to HeLa cells. At this time, it is unknown what biological events determine this antagonistic behavior, however, if in fact similar events occur in the human gut, they may influence the outcome of diarrheic processes simply by determining in which proportions the involved species will compose the intestinal microbiota.

As demonstrated by settling profile assays, EACF 205 and *traA*-positive EAEC strains aggregated after inter-specific recognition during the mid-log phase of growth. Moreover, SEM micrographs showed that the bacterial aggregates were mediated by non-bundle forming, flexible pili. These observations indicated that increased adherence might be mediated by putative F pili expressed by EAEC strains. Endorsing our assumption, inhibition of the putative F pili by zinc significantly reduced the bacterial aggregation and mixed biofilms produced by EAFC 205 and *traA*-positive EAEC strains. SEM images showed that enhanced biofilms formed by cocultures of EACF 205 and *traA*-positive EAEC strains were mediated by pili that promoted bacteria-to-bacteria interactions in addition to adhesion to inert surface. Conversely, biofilms formed by the coculture of EACF 205 and *traA*-negative EAEC strain 17-2 did not display pili and therefore were resistant to zinc treatment.

With regard to biofilms formed by *traA*-positive EAEC strains (Figure 6A), our results are in agreement with a previous report showing that natural F plasmids promoted single biofilm formation by generating cell-to-cell connections mediated by F pili even in F^+^-bacteria populations. Endorsing this idea, it was shown that biofilm formation is also induced by transfer-deficient F plasmids indicating that the phenomenon does not require conjugative DNA transfer itself [20].

Curli fiber displayed by *Enterobacteriaceae *species is an unstable phenotype that is responsive to many environmental conditions. In *C. freundii *and *E. coli *strains, it has been shown that curli fibers mediate the biofilm formation at liquid-solid interfaces [25]. Additionally, the presence of natural F conjugative plasmids in *E. coli *strains was shown to induce the development of mature single biofilms by stimulating the expression of curli fibers after appearance of F pili and following cell-to-cell contact [21]. Based on previously published SEM images [21,25], we were unable to detected curli fibers in single biofilms formed by EACF 205 despite the extensive analysis. Concerning *E. coli *strains, although curli fibers were detected in *traA*-positive EAEC 340-1, their expression was infrequent and incipient either in single biofilms (Figure 6D) or in mixed biofilms formed in the presence of EACF 205 (Figure 6B). Taken together our findings corroborate with previous studies showing the central role of the F pilus in the initial steps of the biofilm formation by *E. coli *strains. Adding to this model, now it is shown that expression of F pili may engage *E. coli *pathotypes in microbial consortia associated with diarrhea.

Zinc is a vital micronutrient in humans and its dietary deficiency occurs worldwide, particularly in developing countries. Numerous studies have suggested that zinc-deficient populations presented an increased risk of contracting diarrhea. Consequently, the zinc administration has been recommended as an additional approach for the prevention and management of diarrhea, being more efficient in treating persistent diarrhea rather than acute cases [38,39]. Although the success of zinc therapy has been widely reported, the mechanisms by which zinc exerts its antidiarrheal effects is not fully elucidated [38,39]. The present results showed that zinc frequently inhibited biofilms formed by typical EAEC strains isolated from diarrheic children, indicating a possible explanation for its efficient use in the management of diarrhea.

## Conclusions

Previously, we reported that typical EAEC strains negative for the AAF fimbriae were statistically associated with persistent diarrhea [9], indicating the occurrence of other adhesion factors among wild-type typical EAEC strains. Here, the results indicate that putative F pili may work as central adhesion factor during the biofilm formation by typical EAEC strains. Moreover, putative F pili engage typical EAEC strains in forming mixed biofilms increasing the overall bacterial adhesion when diarrhea-isolated aggregative *C. freundii *is present.

## Methods

### Bacterial strains

During a case-control study focusing on the epidemiology of EAEC [9], the biofilm-forming aggregative *C. freundii *(EACF) strain 205 was isolated from a child (aged 13 months) on the fifth day of a mucous diarrhea that presented, on average, 15 evacuations per day. A typical EAEC strain was isolated concomitantly from the same child (strain 205-1, genotype CVD432^+^AggR^+^AAF-I^+^PilS^-^Pic^+^). The diffusely adherent *C. freundii *strain 047 was isolated from a healthy child (aged 21 months) together with the atypical EAEC strain 047-1 (CVD432^-^AggR^-^AAF^-^PilS^-^Pic^+^). Typical EAEC strain 340-1, which shares with EAEC 205-1 the same genotype (CVD432^+^AggR^+^AAF-I^+^PilS^-^Pic^+^), was isolated from a persistent (lasting ≥ 14 days) mucous diarrhea affecting a child aged 3 months. This strain was chosen based upon its shared genotype with EAEC 205-1. Forty three typical EAEC strains negative for the AAF alleles I and II and isolated during the same study from children up to 5 years of age were used to evaluate the role of putative pili F and the effect of zinc on the single biofilm formation. Prototype EAEC strains 042 [40] and 17-2 [41] were also used for the assays. Bacterial strains were preserved at -20°C in Luria Bertani (LB) broth with 15% glycerol. Unless otherwise stated, bacterial strains were cultured in LB broth at 37°C for 18 h with constant agitation (200 rpm).

### Primers and PCR conditions

Primers were designed in order to detect multiple alleles of the *agn43 *gene. *Agn43-oxy *primers detect alleles harbored by prototype strains of *E. coli *K12 (Genbank accession numbers: NC_000913, AC_000091, NC_010473 and NC_012759) whose transcription is under the control of the *oxyR *locus. The forward primer (5'-CGATCGATAAGCTAATAATAACC-3') targets the locus *oxyR *(nucleotide position 2069371..2069393 in the Genbank sequence NC_000913) while the reverse primer (5'-GAAGACCACCACTGGTGACA-3') recognizes the region encoding α^43 ^subunit (position: 2069903..2069922). Additionally to *agn43-oxy *primers, oligonucleotides were designed to detect *agn43*-like loci harbored by uropathogenic *E. coli *strains (Genbank sequences NC_004431 and NC_008253) and by *Shighella flexneri *strains (Genbank sequences NC_004337 and NC_004741). The *agn43 *primers (5'-CGTGGATGATGGCGGAAC-3' and 5'-CACCGTTAATGGCTTCAACC-3') amplify a 920 bp fragment spanning the regions that encode the α^43 ^and β^43 ^subunits (position 3492898..3493817 in Genbank NC_004431).

The presence of putative pCTX-like plasmids was investigated employing primers designed to target consensus sequences displayed in the GenBank sequences AF550415 (pCTX-M3 plasmid from *C. freundii*), EU938349 (pCTXM360 plasmid from *K. pneumoniae*) and AY422214 (pEL60 plasmid from *Erwinia amylovora*). On basis of these sequences, the *traJ *primers (5'-AATACCGCTATCCAGCTAAGAG-3' and 5'CCCACTTGCTGTAATCAACG-3') generate an amplicon with 517 bp in length (position 35550..36312 in the sequence AF550415).

Primers *tra *were designed based on the conserved sequences of the *traA *family genes. In relation to the prototype F pilus (Genbank: K01147), the forward primer (5'-AAGTGTTCAGGGTGCTTCTG-3') target the *traA *signal sequence (position: 1940..1959) while the reverse primer (5'-TATTCTCGTCTCCCGACATC-3') recognize the beginning of the *traL *gene (position: 2305..2324). *traA *primers detect the subtypes I (encoded by ColVBtrp and F plasmids), IIa (ColB2), IIb (R124), III (R1) and IV (R100) of the *traA *genes harbored by IncF plasmids [42,43]. Cycling conditions for PCR were as follows: 30 cycles of 94°C for 60 s, 60°C for 60 s, and 72°C for 90 s. Specific EAEC molecular markers as well as virulence factors for other *E. coli *pathotypes were detected using the primers listed in table 1[5,9,14,44-48]. Supernatants derived from bacterial suspensions treated by boiling were used as the source of DNA.

### HeLa cells and infection assays

HeLa cells were cultured in DMEM (Dulbecco's modified Eagle's medium; Gibco BRL) with 10% fetal bovine serum (FBS) and antibiotics (ampicillin [120 μg/mL] and streptomycin [100 μg/mL]) under atmosphere with CO_2 _(4%) at 37°C [49].

For qualitative mixed infection assays, HeLa cells (0.6 × 10^5 ^cells/mL) were cultured on glass coverslips (10 × 10 mm) using 24-well culture plates (600 μL/well) (Costar). Cells were grown to 50%-70% confluence, and the medium was changed to DMEM supplemented with 1.4% mannose (DMEM-mannose) without FBS. For quantitative mixed infection assays, HeLa cells (0.8 × 10^5 ^cells/mL) were cultured in similar way using 12-well culture plates without glass coverslips.

In order to carry out the adhesion assays, HeLa cells were infected with 150 μL of an overnight bacterial culture for three hours at 37°C. After infection, the coverslips were washed five times with Dulbecco's PBS (D-PBS), and the cells were fixed with methanol, stained with May-Grünwald and Giemsa stains, and analyzed using light microscopy. EAEC prototype strain 042 was used as the positive control for the aggregative phenotype.

Qualitative mixed infection assays were performed with two infection steps. Initially, *C. freundii *strains (50 μL of an overnight culture) were used to infect the cells for one hour (preinfection). After this time, *E. coli *strains (150 μL of an overnight culture) were added to the well to begin the coinfection period that lasted 2 h. The coverslips were washed, fixed and stained as described above.

Quantitative mixed infection assays were carried out counting the colony-forming units (CFU). The infection steps were performed in the same way as described for the qualitative assays. After the coinfection step, wells were washed five times with D-PBS and HeLa cells and bacteria were suspended in 0.5% Triton X100 in PBS (2 mL/well). The suspension was then diluted 1:1000 in PBS and 50 μL of the suspension was plated onto MacConkey agar plates. CFU counting was carried out determining the number of lactose-fermenting colonies (*E. coli*) and non-fermenting colonies (*C. freundii*). At least three independent assays were performed with each bacterial suspension plated in triplicate.

### Adhesion assays using pre-conditioned medium

Pre-conditioned DMEM-mannose media were used to verify the role of chemical signals in the studied events. Employing polycarbonate permeable inserts (Transwell^®^-Corning) with high pore density (10^8 ^per cm^2^) the plate wells were separated into two compartments; the upper compartment was loaded with 100 μL of DMEM-mannose, and the lower compartment with 400 μL. The medium was pre-conditioned inoculating the upper compartment with 100 μL of overnight bacterial culture for two hours at 37°C. Thereafter, HeLa cells, in the lower compartment, were infected with 150 μL of 18-h culture of the tested bacteria for two hours at 37°C. Finally, the cells were washed, fixed and stained as described above.

### Pre-conditioned HeLa cells

Adherence assays were also performed employing HeLa cells pre-conditioned by the initial adhesion of *C. freundii *strains. Briefly, host cells were pre-infected with an overnight culture (50 μL) for two hours, treated with gentamicin [200 μg/mL] for one hour, and then washed several times with D-PBS in order to remove the adherent bacteria. Afterwards, pre-conditioned HeLa cells were employed to test the adhesion of EAEC strains using 150 μL of an overnight culture for two hours at 37°C.

### Bacterial aggregation assay

Five milliliters of DMEM-mannose in 10 mL test tubes were inoculated with 10 μL of overnight bacterial cultures (or 5 μL of each bacterial culture when the bacterial aggregation of cocultures were tested) and incubated at 37°C for 18 h without shaking. Afterwards, the optical density at 600 nm (OD_600 nm_) of the culture upper layer (700 μL) was determined for the standing and homogenized culture. Bacterial aggregation was evaluated using the following rate: 1- (standing culture OD/homogenized culture OD).

### Bacterial settling profile

In order to verify the differences in bacterial aggregation, settling assays were carried out to follow bacterial settling kinetics. Settling assays were conducted with DMEM-mannose (1.4%) using overnight or mid-log phase culture (OD_630 nm_: 0.6-0.8). Cultures were homogenized and 700 μL of the monoculture, coculture or mixed monoculture (350 μL of each culture) were placed into cuvettes (1 cm light path) and maintained static in the spectrophotometer in order to register the OD decline. For observation of structures involved in bacterial aggregation, 10 μL of bacterial suspension at the onset of the settling curve (15 min) were deposited on poly-L-lysine-coated coverslips (Thermanox™), fixed with 10 μL of Karnovsky's solution (2.5%. paraformaldehyde, 2% glutaraldehyde in 0.1 M cacodylate buffer, pH 7.4) and processed for scanning electron microscopy analyses as described below.

Ou and Anderson [19] demonstrated that nonlethal concentrations of zinc inhibit the formation of mating pairs and consequential bacterial aggregation by blocking the F-pili adsorption site. To evaluate the action of zinc and magnesium on settling profiles, these chemicals were added (up to a final concentration of 1 mM) to the bacterial culture (1 mL). After 1 min, treated bacteria were pelleted (3.000 *g *for 3 min) and the DMEM-mannose medium was replaced. After resuspending the bacterial pellet, the OD decline was registered as described above. The sulfate heptahydratate form (Fisher) of each tested chemical in sterile aqueous solution was used as stock solution (0.1 M).

### Biofilm formation on glass coverslips

In order to evaluate the development of mixed biofilms supported by *C. freundii *and EAEC strains, biofilm assays were performed using glass coverslips (20 × 20 mm) as adhesion surface that were positioned vertically into 30-mL containers (Sterilin^®^) containing 15 mL of DMEM-mannose. Five microliters of each tested bacterial culture were used to inoculate the medium. Alternatively, control assays based on single biofilm formation were conducted using 10 μL of overnight bacterial culture as inoculum. The containers were incubated at an inclined position (45°) under agitation (170 rpm) for 18 hours at 37°C. Afterwards, the coverslips were washed with PBS, and the biofilms were fixed with methanol, stained with crystal violet (CV) (0.1% aqueous solution) and air-dried for 3 h. Inhibition assays employing zinc (0.25 mM ZnSO_4 _in DMEM-mannose) were conducted in the same way. To quantify the formed biofilms, stained coverslips were accommodated into wells of culture plates (6-well plates) and the optical absorbance (630 nm) generated by biofilm-bound dye was measured using a microplate reader (ELX800™ Absorbance Microplate Reader, Bio-Tec). Both faces of the coverslips were analyzed using optical and scanning electron microscopy.

### Biofilm screening assay and zinc inhibition

In order to screen the biofilm formation of several EAEC strains isolated from children, 96-well flat-bottom polystyrene plates were used [50]. Briefly, 200 μL per well of DMEM-mannose were inoculated with 5 μL of overnight bacterial culture, and then, the plates were incubated overnight at 37°C without shaking. Afterwards, the formed biofilms were stained with CV (15 min), washed once with 200 μL of PBS and air-dried for 3 h. The absorbance (OD at 630 nm) reached by CV adsorbed on the well bottom was determined, and afterwards the bacterium-bound dye was released by the addition of ethanol (200 μL/well). One hundred and fifty microlitres of CV-ethanol solution were transferred to new 96-well plates and the OD_630 nm _was determined. The mean of the absorbances was used as measure of the formed biofilms. Assays focusing on biofilm inhibition were conducted in the same way using DMEM-mannose containing 0.25 mM ZnSO_4_.

### Scanning electron microscopy (SEM)

For SEM observations, samples were processed following standard protocols. Briefly, the samples were fixed overnight at 4°C in Karnovsky's solution (2.5%. paraformaldehyde, 2% glutaraldehyde in 0.1 M cacodylate buffer, pH 7.4) and then were post-fixed with 0,1 M cacodylate buffer (pH 7.4) containing osmium tetroxide (1%) and potassium ferricyanide (0.8%) for 1 h at room temperature. Afterward, the samples were dehydrated in a graded acetone series (30-100%), dried at critical point using CO_2 _as the transition fluid, and sputter-coated with gold (2 min).

### Statistical analyses

Statistical analyses were performed using the software SPSS 13.0. Means were compared using independent-sample T test taking into consideration the Levene's test. Analysis of frequency data was performed employing two-tailed Fisher's exact test. The results with *P *≤ .05 were considered statistically significant.

## Competing interests

The authors declare that they have no competing interests.

## Authors' contributions

ALP conceived the study and designed the experiments. ALP and TNS performed experiments and analyzed data. ALP and LGG wrote the manuscript and were responsible for concepts, vision and direction for the study. ACMMG and ACGA carried out the electron microscopy and image acquisition. All authors read and approved the final manuscript.
